# Intraoperative Corticosteroids and Pacemaker Implantation After Transcatheter Aortic Valve Replacement

**DOI:** 10.7759/cureus.56824

**Published:** 2024-03-24

**Authors:** Catarina Tiago, Marta Dias Vaz, Ana Marques, Melanie Barata, José Pedro Braga, Ana Boa, Ana Filipa Carvalho

**Affiliations:** 1 Anesthesiology, Centro Hospitalar de Vila Nova de Gaia - Espinho, Porto, PRT; 2 Cardiology, Centro Hospitalar de Vila Nova de Gaia - Espinho, Porto, PRT

**Keywords:** vascular system injuries, transcatheter aortic valve replacement, pacemaker, cardiac dysrhythmia, glucocorticoids

## Abstract

Background: Transcatheter aortic valve replacement (TAVR) has revolutionized the treatment of aortic stenosis. However, conduction disturbances leading to pacemaker implantation remain a common complication, increasing morbidity and mortality in these patients. Hence, measures to lower its incidence should be taken, and corticosteroid therapy could be effective by reducing inflammation caused by direct mechanical trauma to the conduction system.

Methods: A retrospective cohort study was conducted at the Centro Hospitalar de Vila Nova de Gaia/Espinho, analyzing the medical records of patients with native severe aortic stenosis who underwent transfemoral TAVR in 2022. The Chi-square test was used to compare the rate of pacemaker implantation in patients who received corticosteroids with patients who didn't. The statistical significance was considered for a p-value <0.05.

Results: A total of 341 patients were included in this study. Monitored anesthesia care was the preferred anesthetic technique (99.1%). Sixty-three point three percent (63.3%) of patients received corticosteroids at the beginning of the procedure. Corticosteroid administration did not significantly affect the incidence of permanent pacemaker implantation (p=0.277), vascular complications on the access site (p=0.765), or in-hospital mortality (p=0.909). Male gender, 1st-degree atrioventricular block, and right branch block were the only identified predictors of permanent pacemaker implantation after transfemoral TAVR (p=0.041 <0.001 and <0.001, respectively).

Conclusion: Corticosteroid administration at the beginning of TAVR doesn’t seem to influence the incidence of permanent pacemaker implantation, which can suggest that other factors play a more important role in the development of conduction disturbances leading to pacemaker implantation.

## Introduction

Aortic stenosis is the most common valvular heart disease in the developed world, and transcatheter aortic valve replacement (TAVR) has revolutionized its management in severe cases and is now the most common treatment in many countries [1]. Nevertheless, the procedure is frequently associated with conduction disturbances, particularly new-onset left bundle branch block and advanced atrioventricular block necessitating permanent pacemaker implantation, which remains the most common complication of this procedure. Despite improvements in technology and operator expertise, its incidence remains high (2-51%) [2].

Pacemaker implantation after TAVR is associated with a higher risk of mortality and rehospitalization for heart failure, which supports the need for the implementation of procedural and possibly pharmacological strategies to reduce the incidence of conduction abnormalities [3]. Although there are some identified risk factors for pacemaker implantation, such as preoperative conduction disturbances and type of prosthesis, the cause of complete atrioventricular block after TAVR is unknown [4-5]. However, due to the anatomical proximity between the aortic valve and the atrioventricular node as well as the His bundle, direct mechanical trauma produced by valve prostheses, catheters, or wires may cause local inflammation, edema, or ischemia and damage to the cardiac conduction system [6]. Furthermore, the atrioventricular block is often transient, which led to the hypothesis that the use of corticosteroids prior to the procedure could reduce tissue edema and fibrosis related to inflammation and hence the incidence of conduction disturbances and pacemaker implantation [7-8].

In this study, we aim to determine if the intraoperative administration of corticosteroids as part of the anesthetic plan is associated with a reduction in pacemaker implantation rate after transfemoral TAVR. Our secondary objective is to describe patient, anesthesia, and procedure characteristics.

## Materials and methods

Study design and population

A retrospective cohort study was conducted at the Centro Hospitalar de Vila Nova de Gaia - Espinho, analyzing the medical records of patients with native severe aortic stenosis who underwent transfemoral TAVR between January 2022 and December 2022. Patients with a previously implanted pacemaker were excluded from the study.

Data collection and measurements

Patient, procedure, and anesthesia details were collected from electronic clinical processes, anesthesia, and/or nursing records. Intraoperative corticosteroid administration was defined as the intravenous administration of at least 125 mg of methylprednisolone (or equivalent) at the beginning of the procedure. Pacemaker implantation was defined as the need for permanent pacemaker implantation in the first 48 hours after the procedure. Vascular complications were defined as arterial dissection, pseudoaneurysm formation, and hematoma requiring surgical drainage on the access site [9].

Statistical analysis

Statistical analysis was performed using SPSS Statistics version 25.0 software (IBM Corp. Released 2017. IBM SPSS Statistics for Windows, Version 25.0. Armonk, NY: IBM Corp.). Categorical variables are presented as frequencies and percentages, and continuous variables as means and standard deviations, or medians and interquartile ranges for variables with skewed distributions. The normal distribution was checked using skewness and kurtosis. Chi-square or Mann-Whitney tests were used to compare groups. The statistical significance was considered for a p-value <0.05.

Ethical considerations

This study was approved by the Centro Hospitalar de Vila Nova de Gaia/Espinho Ethics Committee on March 14, 2023 (approval number: 38/2023-1).

## Results

Between January 2022 and December 2022, 378 patients with native severe aortic stenosis were submitted to transfemoral TAVR at Centro Hospitalar de Vila Nova de Gaia - Espinho. Thirty-seven patients were excluded due to previous pacemaker implantation. Out of the 341 patients included in this study, 53.7% were female, and the median age was 81±10 years. The risk of mortality after surgical aortic valve replacement estimated by the Society of Thoracic Surgeons score was 3.02±1.94%. The procedures lasted about 72±30 minutes and monitored anesthesia care (MAC), which was the anesthetic technique performed in the majority of cases (99.1%). Self-expanding valves were more commonly used than balloon-expandable valves (70.4% and 29.6%, respectively). Sixty-three point three percent (63.3%) of patients received a corticosteroid at the beginning of the procedure. After percutaneous TAVR, patients remained in a high dependency unit for about 1±1 days, and the total length of hospital stay was 5±2 days. The permanent pacemaker implantation rate was 18.2%, vascular complications on the access site were seen in 5.9% of patients, and in-hospital mortality was 0.9%.

Patient, procedure, and anesthesia details are described in Table 1. Patients who received corticosteroids were significantly older (p=0.001), had chronic obstructive pulmonary disease (COPD) less frequently (p=0.047), and received a self-expanding valve less frequently (p=0.048).

**Table 1:** Patient, procedure, and anesthesia details AV: atrioventricular, CAD: coronary artery disease, CKD: chronic kidney disease, COPD: chronic obstructive pulmonary disease, IQR: interquartile range, MAC: monitored anesthesia care, STS: Society of Thoracic Surgeons

	Corticosteroids (n=216)	No corticosteroids (n=125)	p-value
Patient details			
Male gender - n(%)	103 (47.7)	55 (44.0)	0.511
Age (years) - median (IQR)	82 (10)	79 (12)	0.001
STS score - median (IQR)	3.03 (1.94)	2.98 (1.98)	0.805
Hypertension - n (%)	191 (88.4)	107 (85.6)	0.449
Diabetes mellitus - n (%)	92 (42.6)	54 (43.2)	0.913
Dyslipidemia - n (%)	188 (87.0)	100 (80.0)	0.084
Obesity - n (%)	58 (26.9)	28 (22.4)	0.362
CAD - n (%)	95 (44.0)	43 (34.4)	0.082
Ejection fraction - median (IQR)	57 (8)	55 (11.5)	0.096
Atrial fibrillation - n (%)	43 (19.9)	32 (25.6)	0.221
First-degree AV block - n (%)	42 (19.4)	16 (12.8)	0.116
Right branch block - n (%)	22 (10.2)	12 (10.3)	0.862
Left branch block - n (%)	47 (21.8)	17 (13.6)	0.063
COPD - n (%)	36 (16.7)	32 (25.6)	0.047
CKD - n (%)	76 (35.2)	37 (29.6)	0.291
Stroke - n (%)	17 (7.9)	12 (9.6)	0.581
Procedure details			
Duration (minutes) - median (IQR)	74 (30)	71 (28)	0.144
Self-expanding valve - n (%)	144 (66.7)	96 (76.8)	0.048
Anesthesia details			
MAC - n (%)	213 (98.6)	125 (100)	0.186

Patients who received a corticosteroid had a higher incidence of permanent pacemaker implantation (19.9% vs. 15.2%), but there were no statistically significant differences between groups (p=0.277). Length of stay in a high dependency unit, total length of stay, vascular complications on the access site, and in-hospital mortality didn’t differ between groups (Table 2). Mortality was due to cardiovascular causes in only one out of the three cases.

**Table 2:** Outcomes after TAVR HDU: high dependency unit, IQR: interquartile range, LOS: length of stay

	Corticosteroids (n=216)	No corticosteroids (n=125)	p-value
LOS in HDU (days) - median (IQR)	1 (1)	1 (1)	0.806
Total LOS (days) - median (IQR)	5 (3)	6 (2)	0.421
Pacemaker implantation - n (%)	43 (19.9)	19 (15.2)	0.277
Vascular complications - n (%)	12 (5.6)	8 (6.4)	0.765
In-hospital mortality - n (%)	2 (0.9)	1 (0.8)	0.909

Table 3 describes the predictors of permanent pacemaker implantation after percutaneous TAVR. Among patients who needed pacemaker implantation, male gender, and previous first-degree atrioventricular block or right branch block were the only predictors identified in this study (p=0.041, <0.001 and <0.001, respectively).

**Table 3:** Predictors of pacemaker implantation AV: atrioventricular, IQR: interquartile range, STS: Society of Thoracic Surgeons

	Pacemaker (n=62)	No pacemaker (n=279)	p-value
Male gender - n (%)	36 (58.1)	122 (43.7)	0.041
Age (years) - median (IQR)	82 (9)	81 (11)	0.153
STS score - median (IQR)	2.78 (2.34)	3.04 (1.93)	0.359
Atrial fibrillation - n (%)	15 (24.2)	60 (21.5)	0.644
First-degree AV block - n (%)	21 (33.9)	37 (13.3)	<0.001
Right branch block - n (%)	14 (22.6)	20 (7.2)	<0.001
Left branch block - n (%)	17 (27.4)	47 (16.8)	0.054
Self-expanding valve - n (%)	41 (66.1)	199 (71.3)	0.418

## Discussion

In our study, MAC was the most commonly used anesthetic technique, and this finding is in accordance with other European and North American centers [10]. Although studies suggest there are no differences between general anesthesia and MAC in terms of mortality or major complications after TAVR, MAC appears to be associated with shorter procedure duration as well as intensive care and hospital length of stay [11-12]. This choice could be further explained by the fact that patients are usually old with multiple comorbidities.

Permanent pacemaker implantation following percutaneous TAVR occurred in 18.2% of our patients, which is within the range reported in a meta-analysis by Xi et al. (6.2-32.8%) and similar to the results from the SWEDEHEART observational study (14.1%) and data from the German Aortic Valve Registry (15.1%) [13-15]. However, corticosteroid administration at the beginning of the procedure didn’t reduce the incidence of pacemaker implantation, as opposed to the findings described by Oestreich et al. in their study [8]. Our finding is in agreement with the results presented by Bernhard et al. in a large prospective cohort study, in which patients who received corticosteroids at least one week prior to the procedure didn’t have a lower risk of atrioventricular conduction delays leading to pacemaker implantation [16]. A prospective study conducted by Chenna et al. also showed that patients treated either with chronic or acute corticosteroids before TAVR didn’t have lower permanent pacemaker implantation rates [17]. The CORTAVI study, which retrospectively analyzed patients who were exposed to glucocorticoids after TAVR, didn’t find a reduction in the frequency of permanent pacemaker implantation, atrioventricular block, right bundle branch block, or left bundle branch block [18]. These results may be explained by the presence of other factors that influence the occurrence of conduction disturbances, such as inter-individual heterogeneity in the anatomical location of the auriculoventricular node and the bundle and implantation depth of the prosthesis that can't be altered with corticosteroid therapy [19-20]. These findings may suggest that conduction disturbances leading to pacemaker implantation are primarily caused by direct mechanical trauma to the conduction system and that local inflammation and edema play a minor role in the development of this major complication of TAVR. Nonetheless, corticosteroid administration didn't increase the rate of vascular complications on the access site previously suggested by Fink et al., nor did it increase in-hospital mortality, which is in accordance with a meta-analysis conducted by Macedo et al. [21-22].

Although corticosteroid administration wasn’t associated with a permanent pacemaker implantation rate, we found other predictors, namely male gender, first-degree atrioventricular block, and right bundle branch block. These findings are in agreement with other studies, although age, left bundle branch block, and self-expanding valve type are also associated with a higher incidence of pacemaker implantation [23-25].

The retrospective character of the study is one of its major limitations. Furthermore, it is a single-center study. Future studies evaluating the appropriate timing, dose, and effect of maintaining corticosteroid therapy after the procedure are needed.

## Conclusions

In this study of patients with native severe aortic stenosis subjected to transfemoral TAVR, MAC was the most commonly used anesthetic technique. Corticosteroid administration at the beginning of the procedure didn’t influence the rate of permanent pacemaker implantation, the incidence of vascular complications at the access site, or in-hospital mortality. Male gender and previous first-degree atrioventricular block or right branch block were the only predictors of pacemaker implantation identified in this study.
